# A 5-Lipoxygenase Inhibitor, Zileuton, Modulates Host Immune Responses and Improves Lung Function in a Model of Severe Acute Respiratory Syndrome (SARS) Induced by *Betacoronavirus*

**DOI:** 10.3390/v15102049

**Published:** 2023-10-04

**Authors:** Rafaela das Dores Pereira, Rayane Aparecida Nonato Rabelo, Natália Fernanda de Melo Oliveira, Samuel Luiz Teixeira Porto, Ana Claudia dos Santos Pereira Andrade, Celso M. Queiroz-Junior, César Luís Nascimento Barbosa, Luiz Pedro de Souza-Costa, Felipe Rocha da Silva Santos, Fernando Bento Rodrigues Oliveira, Bárbara Luísa Vieira da Silva, Hanna L. Umezu, Raquel Ferreira, Glauber S. F. da Silva, Jader Santos Cruz, Mauro Martins Teixeira, Vivian Vasconcelos Costa, Fabiana Simão Machado

**Affiliations:** 1 Department of Biochemistry and Immunology, Institute of Biological Sciences, Federal University of Minas Gerais, Belo Horizonte 31270-901, MG, Brazil; rafaelapereiranutri@gmail.com (R.d.D.P.); rayane.rabelo10@gmail.com (R.A.N.R.); melonataliaf@gmail.com (N.F.d.M.O.); samuel.porto192@gmail.com (S.L.T.P.); souzaluizpedro@gmail.com (L.P.d.S.-C.); felipe.rocha1@live.com (F.R.d.S.S.); fernandobento421@gmail.com (F.B.R.O.); ferreiraquel93@gmail.com (R.F.); jadercruzytrio@gmail.com (J.S.C.); mmtex.ufmg@gmail.com (M.M.T.); 2 Department of Morphology, Institute of Biological Sciences, Federal University of Minas Gerais, Belo Horizonte 31270-901, MG, Brazil; anaclaudiaandrade29@gmail.com (A.C.d.S.P.A.); cmqj@yahoo.com.br (C.M.Q.-J.); barbaraluisa.ufmg@gmail.com (B.L.V.d.S.); 3 Program in Health Sciences: Infectious Diseases and Tropical Medicine, Interdisciplinary Laboratory of Medical Investigation, Faculty of Medicine, Federal University of Minas Gerais, Belo Horizonte 31270-901, MG, Brazil; 4 Department of Physiology and Biophysics, Institute of Biological Sciences, Federal University of Minas Gerais, Belo Horizonte 31270-901, MG, Brazil; hanna.umezu@gmail.com (H.L.U.); glauber@ymail.com (G.S.F.d.S.)

**Keywords:** zileuton, viral infection, MHV, SARS-CoV-2, COVID-19

## Abstract

Exacerbated inflammatory responses are a hallmark of severe coronavirus disease 2019 (COVID-19). Zileuton (Zi) is a selective inhibitor of 5-lipoxygenase, an enzyme involved in the production of several inflammatory/pro-resolving lipid mediators. Herein, we investigated the effect of Zi treatment in a severe acute respiratory syndrome (SARS) model. Mouse hepatitis virus (MHV)3-infected mice treated with Zi significantly improved the clinical score, weight loss, cardiopulmonary function, and survival rates compared with infected untreated animals. The protection observed in Zi-treated mice was associated with a lower inflammatory score, reduced dendritic cell-producing tumor necrosis factor (TNF), and increased neutrophil-producing interleukin (IL)-10 in the lungs three days after infection (dpi). At 5 dpi, the lungs of treated mice showed an increase in Th2-, Treg CD4^+^-, and Treg CD8^+^-producing IL-10 and reduced Th1 infiltrating cells. Furthermore, similar results were found upon Zi treatment after SARS-CoV-2 infection in transgenic mice expressing the human angiotensin I-converting enzyme 2 (ACE2) receptor driven by the cytokeratin-18 (K18) gene promoter (K18-hACE2), significantly improving the clinical score, weight loss, and lung inflammatory score compared with untreated animals. Our data suggest that Zi protects against developing severe lung disease during SARS induced by betacoronavirus without affecting the host’s capacity to deal with infection.

## 1. Introduction

The disease caused by severe acute respiratory syndrome coronavirus 2 (SARS-CoV-2) is highly transmissible and potentially fatal. SARS-CoV-2 infects the upper and lower airways, causing mild symptoms such as fever, cough, fatigue, runny nose, and sneezing, and can evolve with severe symptoms, such as severe acute respiratory syndrome (SARS), hypoxemic respiratory failure, shock, multiple organ failure, and death [1]. Evidence suggests that the exacerbated inflammatory process induced by the SARS-CoV-2 infection plays a key role in the severity of COVID-19 and mortality [2,3,4]. Severe COVID-19 is associated with high levels of circulating mediators, including pro- and anti-inflammatory cytokines and lipid mediators; profound lymphopenia; and substantial infiltration of mononuclear cells into the lungs, heart, spleen, lymph nodes, and kidneys [5,6,7].

Recent studies have shown that critically ill patients with SARS-CoV-2 infection present dysregulated inflammatory responses, including lipid mediator production [8,9,10,11]. Thus, the modulation of lipid mediators may be a therapeutic target for SARS-CoV-2. Among these lipid mediators, leukotrienes (LTs) and lipoxins (LXA) have been highlighted because they can affect prognosis and play a dual role in the pathogenesis of several chronic inflammatory diseases, including bacterial, parasitic, and viral infections in experimental models [12,13,14]. Zileuton (Zi) is a selective inhibitor of the enzyme 5-lipoxygenase (5-LO), which is involved in intracellular pathways that trigger the production of both LTs and LXA. Zi is approved for use in patients with asthma [15,16].

We hypothesized that Zi could have a protective effect against betacoronavirus-induced diseases. Here, we first used a model of mouse hepatitis virus (MHV)-3 infection, a murine betacoronavirus that emulates severe COVID-19 in mice [17]. Zi treatment significantly improved clinical scores, weight loss, cardiopulmonary function, and survival rates compared with vehicle-treated infected littermates. Zi-treated mice showed reduced inflammatory scores in the lungs, reduced dendritic cell-producing tumor necrosis factor (TNF), and increased neutrophil-producing interleukin (IL)-10 in the lungs 3 days after infection (dpi), the peak of lung damage. At 5 dpi, the peak of systemic disease manifestation, increased Th2-, Treg CD4^+^-, and Treg CD8^+^-producing IL-10, and reduced Th1 infiltrating cells were observed in the lungs of treated mice. Similar protective effects were observed upon Zi treatment in transgenic mice expressing the human angiotensin I-converting enzyme 2 (ACE2) receptor driven by the cytokeratin-18 (K18) gene promoter (K18-hACE2) infected by SARS-CoV-2. Interestingly, the effects of Zi were independent of the control of viral titers in the lungs.

These findings suggest that Zi treatment controls the exacerbated deleterious inflammatory responses in the lungs of mice infected with both MHV-3 and SARS-CoV-2 without affecting the host’s ability to deal with the infection. Finally, using Zi along with an antiviral drug may be beneficial for treating betacoronavirus-induced diseases.

## 2. Materials and Methods

### 2.1. Cell, Virus, and Plaque Assay

L929 cells purchased from the American Type Culture Collection (ATCC ^®^ CCL-1) were cultured in a controlled atmosphere (37 °C, 5% CO_2_) in Dulbecco’s modified Eagle’s medium (DMEM, Gibco, Gibco, Grand Island, NY, USA) supplemented with 7% fetal bovine serum (FBS, Cultilab, Campinas, Brazil), and 100 U/mL of penicillin (Gibco) and 100 μg/mL of streptomycin (Gibco). The MHV-3 strain was kindly provided and sequenced (GenBank accession number MW620427.1. [18] by Dr. Clarice Arns and Dr. Ricardo Durães-Carvalho from the State University of Campinas (UNICAMP, Brazil), and propagated in L929 cells. For viral titration, 100 μL of serially diluted virus suspension, plasma samples, and tissue homogenates (1:9; tissue: DMEM) were inoculated onto a confluent monolayer of L929 cells grown in 24-well plates. After gentle agitation for 1 h (4 × 15 min), the samples were harvested, and the culture medium was replaced (DMEM containing 1.6% carboxymethylcellulose, 2% FBS, and 1% penicillin-streptomycin-glutamine) and maintained for two days at 37 °C and 5% CO_2_. Then, cells were fixed with 10% neutral-buffered formalin for 1 h and stained with 0.1% crystal violet. Viral titers were determined as plaque-forming units (PFU).

### 2.2. Mouse Strains

C57/BL6 obtained from the Central Animal House at the Federal University of Minas Gerais (UFMG) and hK18 human angiotensin I-converting enzyme 2 (ACE2) purchased from the Jackson Laboratories and maintained at the Immunopharmacology Lab at the Institute of Biomedical Sciences (ICB)/UFMG were housed in individually ventilated cages placed in an animal facility at 24 ± 2 °C on a 12/12 h light/dark cycle, receiving ad libitum access to water and food. All experimental procedures were performed with mixed groups (male and female) of mice aged 6–9 weeks at the NB2 and NB3 facilities and were approved by the Ethics Committee for Animal Experimentation of the UFMG (Approval no 190/2020).

### 2.3. MHV-3 and SARS-CoV-2 Infections

Mice were anesthetized with an intraperitoneal injection of ketamine (Syntec, 50 mg/kg): xylazine (Syntec, 5 mg/kg), and received an intranasal inoculation of 30 μL of loaded or unloaded sterile saline (mock controls) with MHV-3 (3 × 10^3^ PFU) or SARS-CoV-2 (clinical isolate from a nasopharyngeal swab obtained from a confirmed case of COVID-19 in Rio de Janeiro, Brazil, GenBank accession no. MT710714, Gamma variant, 2 × 10^4^ PFU). Symptoms of illness, including goosebumps, arching of the back, weight loss, facial swelling, and lack of activity, were monitored daily for five or ten days post-inoculation (dpi).

### 2.4. Treatment with Zi

For the treatment, mice were divided into three groups: mock, vehicle, and treated. Mock animals received an intranasal inoculation of 30 μL of sterile saline. In contrast, mice in the vehicle and treated groups received an intranasal inoculation of 30 μL loaded with MHV-3 or SARS-CoV-2. Then, 24 h after infection, the treated group received a solution containing 1.5, 3, 15, or 30 mg/kg of Zi, diluted in 100 μL of 0.5% carboxymethylcellulose (CMC) by oral gavage. The treatment was administered orally for ten days, and the animals received the drug every 12 h. The vehicle group received 100 μL of carboxymethylcellulose (CMC) 0.5% for ten days, every 12 h.

### 2.5. Tissue Collection

After anesthesia (ketamine: xylazine, 80 mg/kg:10 mg/kg, i.p.), control (mock), infected, and infected and treated mice at 3 and 5 dpi were euthanized. Blood samples were collected from the abdominal cava vein and placed in tubes coated with ethylenediaminetetraacetic acid (EDTA; BD, São Paulo, Brazil). The lungs were harvested and quickly rinsed with cold, sterile saline, and the right lobes were frozen. The left lobe was fixed by immersion in a 10% neutral buffered formalin solution (Synth).

### 2.6. Hematological Evaluation

The numbers of circulating platelets, leukocytes, red cells, granulocytes, and lymphocytes were determined in blood samples using a Celltac MEK-6500K hemocytometer (Nihon Kohden, Indaiatuba, São Paulo, Brazil).

### 2.7. Histopathology

Formalin-fixed and paraffin-embedded (FFPE) tissues were sectioned into 5-μm thickness slices, stained with hematoxylin-eosin (H&E), and examined under light microscopy. Inflammation-mediated injury in mouse lungs was determined by a pathologist (CMQJ) blinded to the experiment by employing a scoring system encompassing (i) airway inflammation (up to 4 points); (ii) vascular inflammation (up to 4 points); (iii) parenchyma inflammation (up to 5 points); and (iv) general neutrophil infiltration (up to 5 points) [19]. Fundamental pathological lesions, including signs of inflammation (i.e., inflammatory cell infiltration and edema), cell death (i.e., necrosis), tissue hyperplasia, and hemorrhage, were evaluated regarding intensity and extension and graded according to the adapted score.

### 2.8. Cytokine Assay

Lung homogenates were prepared by homogenizing 40 mg of frozen tissue in 400 mL of cold cytokine extraction buffer (100 mM Tris pH 7.4, 150 MM NaCl, 1 mM EGTA, 1 mM EDTA, 1 mM Triton X-100, 1% sodium deoxycholate, 0.5%, and protease inhibitor cocktail 1%. After centrifugation at 14,000× *g* for 15 min at 4 °C, the supernatant was collected, and the concentration of TNF, IFN-γ, IL- 10, IL-6, IL-17, IL-1β, TGF- β, CCL2, and CXCL1 was measured using the mouse DuoSet ELISA System (R&D Systems Inc., Minneapolis, MN, USA) following the manufacturer’s instructions.

### 2.9. Respiratory Mechanics

To measure respiratory system compliance, full-range pressure-volume (PV) curves were constructed similar to those in our previous study [17] and adapted from [20,21]. Briefly, the mice were divided into three groups, and three days dpi, the animals were deeply anesthetized until respiratory arrest. The mice were tracheostomized, and a polyethylene tube (P50) was inserted into the trachea. The PV curve was generated by injecting air volume in a continuous manner using a 3 mL syringe and an automated syringe pump (Bonther, Ribeirao Preto, Brazil) at a rate of 3 mL/min until the intratracheal pressure peaked at approximately 35 cmH_2_O. At peak pressure, the syringe pump was manually switched for the deflation limb, deflated at the same rate until the pressure reached approximately 15 cmH_2_O, and finally inflated again to the resting lung volume [20]. Both volume and pressure signals were acquired and recorded using PowerLab software (LabChart v7, AdInstruments, Sydney, Australia). At least two consecutive sequences of inflation and deflation were performed, and full-range PV curves for each animal were obtained. If leaks or inappropriately high pressures were detected, the animal was excluded from the analysis. Vital capacity was determined by maximum insufflation (lung volume at 35 cmH_2_O), and static compliance of the respiratory system (expressed in mL/cmH_2_O) was measured at the steepest point of the deflation limb of the PV curve [20,21,22].

### 2.10. ECG Recording

A six-channel non-invasive electrocardiograph (InCardio X, Inpulse Animal Health^®^, Florianópolis, Santa Catarina, Brazil) was used. The recordings were made without anesthesia once anesthetic drugs were recognized to influence the ECG markers, as highlighted previously [23]. The study ensured precise positioning by manually restraining the rodents in a dorsal recumbent stance on a plastic-covered wooden table, followed by the application of conductive electrocardiographic gel and attachment of four alligator clip electrodes on the forelimbs and hind limbs, as established previously [24]. All the procedures were performed in a quiet room to mitigate stress. For lead second frontal plane deviation (DII), readings were captured at a standard speed of 100 mm/s, with sensitivity calibrated to 3N.

### 2.11. Flow Cytometry

Mice infected or uninfected with MHV-3 were sacrificed at 3 and 5 dpi. The spleens were removed and processed as previously described [25]. The lungs were removed and processed as previously described [26]. Purified cells from the lungs and spleen were plated and incubated with brefeldin A (10 μg/mL) (Invitrogen, Waltham, MA, USA) for 3 h at 37 °C in the presence of 5% CO_2_. Cells were blocked with Fc Block (antibody CD16/CD32 in PBS/BSA 1%), followed by stain using specific combinations of antibodies for cell surface molecule labeling: CD3, CD11b (APC-Cy7); CD4, Ly6C (PE-Cy7); CD8, Ly6G, SinglecF (BV421); CD25, CD45 (PerCP-Cy5.5); F4/80 (FITC); CD11c (V500), and isotype controls (all from BD Biosciences). For intracellular staining, the following antibodies were added: IFNγ (Alexa 488), IL-17, forkhead box P3 (FOXP3) (PE), TNF (PE), and IL-10 (APC). A total of 30,000 cells (events) were acquired using a FACSCanto II cytometer (Becton, Franklin Lakes, San Jose, CA, USA) and analyzed using the FlowJo software (version 10).

### 2.12. Statistical Analysis

The statistical significance of differences in values between the control and treated/infected groups was assessed using Student’s *t*-test, two-way analysis of variance (ANOVA), with Sidak’s post-test. Differences were considered statistically significant at *p* ≤ 0.05. GraphPad Prism software (version 7.0) was used for statistical analyses.

## 3. Results

### 3.1. Treatment with Zi Improves Survival Rates of MHV-3-Infected Mice Regardless a Control of Viremia

**Figure 1:**
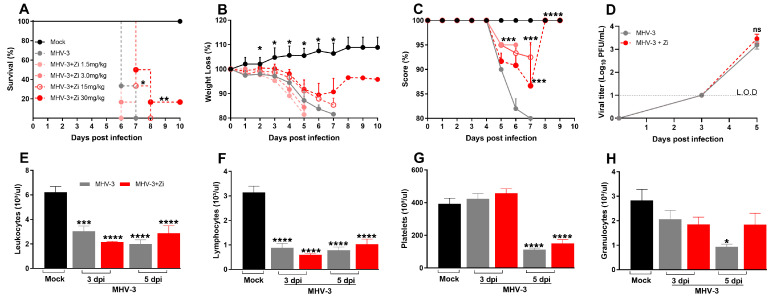
Kaplan–Meier survival curve of control (Mock), untreated MHV-3-infected, and zileuton (Zi) treated MHV-3-infected mice (Zi: 1.5; 3.0, 15, and 30 mg/mL) (**A**). Body weight change after infection was assessed by bidirectional repeated measures analysis of variance (ANOVA) and Sidak’s multiple comparison test. Data are presented as mean ± standard error of the mean (SEM) (**B**). Clinical scoring of control, infected, and Zi-treated infected animals was performed daily until the end of the experiment (**C**). Viral load was determined in plasma collected from mice infected with MHV-3 using the plaque assay and/or infected and treated with Zi (30 mg/mL). Results are presented as log 10 PFU/μL of plasma. Differences between the groups were assessed using the post hoc Kruskal–Wallis and Dunn test (**D**). Differential blood count highlighting leukocytes (**E**), lymphocytes (**F**), platelets (**G**), and granulocytes (**H**), observed on 3 and 5 dpi. Differences between the control, infected, and/or infected-treated groups were assessed by one-way ANOVA and Dunnett’s multiple comparison test. Data are presented as mean ± SEM. LOD, limit of detection. ns, not significant. * *p* < 0.05; ** *p* < 0.01; *** *p* < 0.001; **** *p* < 0.0001. Abbreviations: MHV-3, mouse hepatitis virus 3; PFU, plaque-forming units.

Zi is capable of inhibiting the production of LTs and LXs, which are eicosanoids with pro and anti-inflammatory/pro-resolving actions, respectively. Selective blockade of the main enzyme (5-LO) in the pathway producing these mediators by this drug is a promising target for several diseases, including COVID-19. First, the effect of Zi in an animal model of respiratory coronavirus (MHV-3) infection was investigated through a dose-dependent treatment with Zi (1.5, 3, 15, and 30 mg/kg) (Figure 1A). Animals treated with the highest dose of Zi (30 mg/kg) were able to delay and partially prevent lethality (Figure 1A), reduce weight loss (Figure 1B), and improve clinical scores (Figure 1C) induced by MHV-3-infection, when compared to vehicle-treated animals. Considering that a dose of 30 mg/kg showed the best effects, it was chosen for further experiments. Since the infected animals showed a significant reduction in weight loss from 3 days after infection (dpi) (Figure 1B) and began to succumb to infection at 6 dpi (Figure 1A), we chose to carry out the subsequent analysis at 3 and 5 dpi. To understand how Zi protects mice, the number of plaque-forming units (PFU) in the plasma of animals was verified. Both the infected groups showed elevated viremia. No difference was observed between the Zi- and vehicle-treated groups (Figure 1D), demonstrating that the delay and increased survival rates were not dependent on viremia control. Subsequently, we evaluated the clinical disease. On the third and fifth dpi, hematological analyzes were performed, which showed that the infection caused severe leukopenia (Figure 1E) and lymphopenia (Figure 1F) compared to control animals. Among other parameters, it was observed that on the fifth dpi, there was a reduction in platelets in both groups (Figure 1G), but a reduction in granulocytes was observed only in untreated infected animals (Figure 1H), suggesting that Zi can regulate granulocyte numbers during MHV-3 infection.

### 3.2. Zi Treatment Prevent Lung Tissue Damage Regardless of Viral Load Control

**Figure 2:**
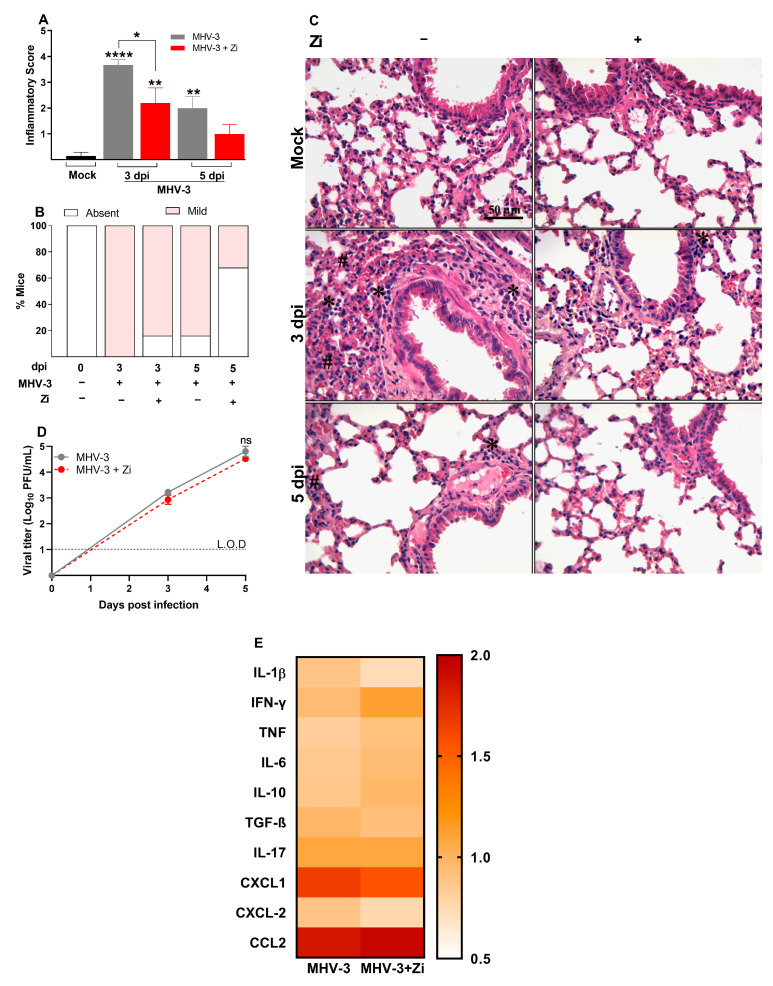
Histopathological evaluation regarding the general inflammatory score. Comparisons between the Mock and infected groups were performed using the post hoc Kruskal–Wallis and Dunn tests (**A**). Percentages of mice according to the degree of inflammatory cell infiltration (**B**). Hematoxylin and eosin (H&E) staining of lung sections showing signs of inflammatory lesion in infected mice (**C**). * foci inflammatory, # hyperplasia, and H hemorrhage. Viral load was determined in lung extracts of mice infected with MHV-3 and/or infected and treated with zileuton (Zi) using the plaque assay. Results are presented as log 10 PFU/g of tissue (**D**). Heat map of cytokines and chemokines concentration levels (fold change) in lung homogenates (**E**). Differences between the groups were assessed using the Kruskal–Wallis and Dunn post hoc tests. LOD, limit of detection. ns, not significant. * *p* < 0.05; ** *p* < 0.01; **** *p* < 0.0001.

To observe the effect of Zi treatment on lung tissue, histopathological analyses were performed at 3 and 5 dpi. The results demonstrated that at 3 dpi, there was more pronounced tissue damage than at 5 dpi in both groups (Figure 2A); however, treatment with Zi significantly reduced the inflammatory scores at 3 dpi when compared to the infected and vehicle-treated groups. At 5 dpi, the Zi-treated group no longer presented a statistically significant difference from the mock-treated group (Figure 2A,C). Moreover, at 3 dpi, 100% of the infected, untreated animals presented with lesions in the lung tissue, whereas this percentage was reduced to 80% in the treated group. At 5 dpi, this difference increased to 80% in the untreated group versus only 30% in the Zi-treated group (Figure 2B). Notably, the amount of PFU was similar in this tissue between the treated and untreated animals on days 3 and 5, demonstrating that the improvement in lung tissue was independent of the number of viral particles present (Figure 2D). Considering that the exacerbated production of inflammatory cytokines is a major hallmark of severe disease, the levels of cytokines in the lung tissue were verified. Zi-treated mice presented at 3 dpi a higher mean level of IFN-γ, TNF, and IL-10 when compared to the untreated group, however, a reduction in CXCL-2, IL-1β, and CXCL-1 was observed (Figure 2E).

### 3.3. Zi Reduces the Severity of Cardiopulmonary Complications during MHV-3 Infection

**Figure 3:**
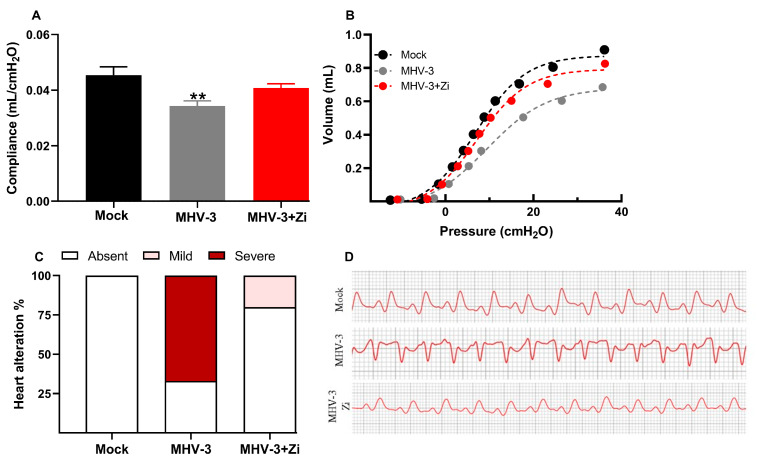
Analysis of compliance of the respiratory system (**A**) in control mice, mice infected with MHV-3 and/or infected and treated with zileuton (Zi). Compliance was calculated from the steepest point of the deflation limb of the pressure-volume (PV) curve. Representative PV curves of animals from each experimental group (mock, MHV-3, and MHV-3+Zi) (**B**). Percentage of cardiac changes in control mice and mice infected with MHV-3 and/or infected and treated with Zi (**C**). Computerized electrocardiogram tracings of control mice and mice infected with MHV-3 and/or infected and treated with Zi. Demonstration of the second frontal plane deviation (DII) records, with a speed of 50 mm/s and amplitude 2 N (**D**). ** *p* < 0.01.

Considering the reduction in inflammation in the lungs, we evaluated whether treatment with Zi would improve the pathophysiological conditions caused by infection. In addition to lung function, cardiac function was also analyzed because the heart is physiologically connected to the lungs and undergoes the consequences of viral infection. Figure 3A,B demonstrated that at 3 dpi, when infected, untreated mice presented a more pronounced lesion in the lungs, a significant reduction in lung compliance was observed compared to the mock group. This effect was observed in the PV curves of representative animals from each experimental group (Figure 3B). Notably, treatment with Zi protected lung function, maintaining the compliance of the respiratory system closer to that of the mock group (Figure 3A,B).

Electrocardiogram analysis revealed that treated animals exhibited minor cardiac alterations, whereas the untreated group displayed pronounced alterations (Figure 3C). Furthermore, Zi treatment helped maintain a heart pattern more closely resembling that observed in the mock animals (Figure 3D).

### 3.4. Zi Treatment Was Associated with an Increased Expansion of IL-10 Producing Neutrophils in the Lungs of MHV-3 Infected-Mice

**Figure 4:**
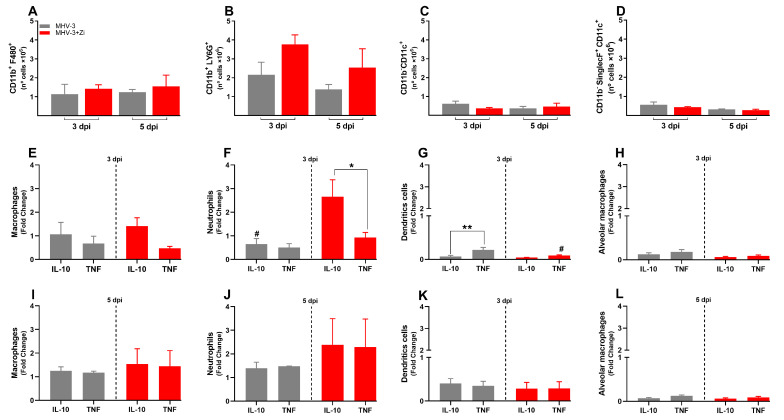
Flow cytometry analyses showing the total cell numbers in the lung at 3 and 5 dpi among groups of macrophages (CD11b+ F480+) (**A**), neutrophils (CD11b+ LY6G+) (**B**), dendritic (CD11b-CD11c+) (**C**), and macrophages alveolar (CD11b+SinglecF+CD11c+) (**D**). Macrophages (**E**,**I**), neutrophils (**F**,**J**), dendritic cells (**G**,**K**), and alveolar macrophages (**H**,**L**)-producing IL-10 and TNF at 3 and 5 dpi. * *p* < 0.05; ** *p* < 0.01; (* representation among the same groups); (# representation between different groups). Abbreviations: IL, interleukin; TNF, tumor necrosis factor.

To better understand the protection of lung function during Zi treatment, the profiles of infiltrating cells in this tissue were investigated. In the lungs, were not observed significant differences in the number of cells of the innate immune response: macrophages (CD11b+F480+, Figure 4A); neutrophils (CD11b+LY6G+, Figure 4B); dendritic cells (CD11b-CD11c+, Figure 4C), and alveolar macrophages (CD11b-SinglecF+CD11c+, Figure 4D). However, the cytokine profiles differed between the untreated and Zi-treated groups. Zi treatment did not alter the numbers of macrophages and alveolar macrophages producing IL-10 or TNF (Figure 4E,H), but resulted in a significant increase in the number of IL-10-producing neutrophils (Figure 4F), and reduced the number of TNF-producing dendritic cells at 3 dpi (Figure 4G). No differences were observed at 5 dpi among these groups (Figure 4K,L).

### 3.5. Treatment with Zi Promotes the Increasing Treg Cells Producing IL-10 into Lung Tissue during MHV-3 Infection

**Figure 5:**
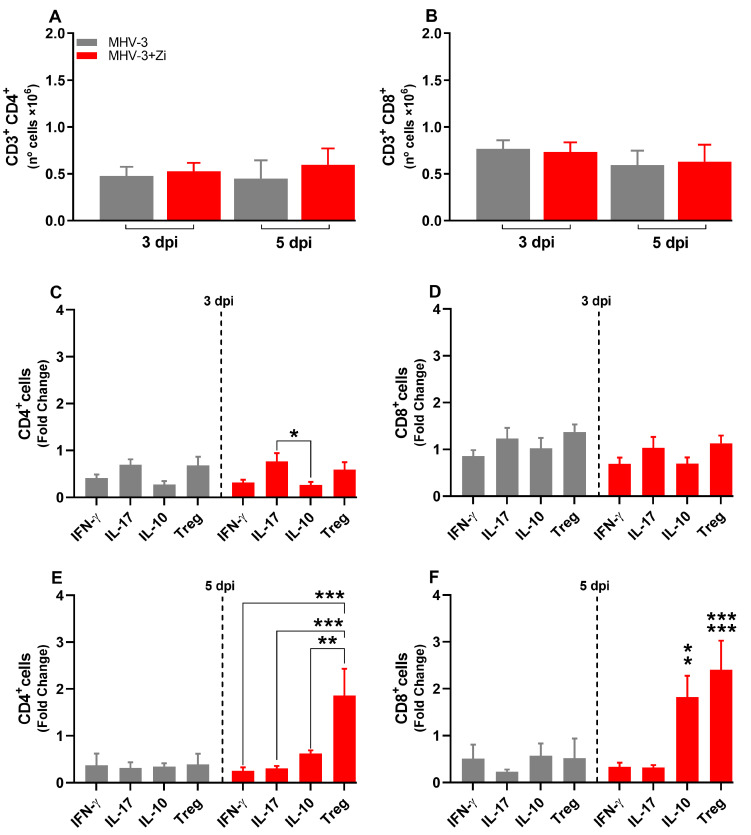
Flow cytometric analyses showing the total cell numbers in the lung at 3 and 5 dpi between the CD3+CD4+ (**A**) and CD3+CD8+ (**B**) groups. CD4+ (**C**,**E**) and CD8+ (**D**,**F**) cells-producing IFN-γ, IL-17, IL-10, and FOXP3^+^ IL-10^+^ (Treg) at 3 and 5 dpi. * *p* < 0.05; ** *p* < 0.01; *** *p* < 0.001. Abbreviations: FOXP3, forkhead box P3; IFN-γ, interferon-gamma; IL, interleukin.

The adaptive immune response is characterized by delayed and specific actions. No difference in the numbers of CD4+ and CD8+ lymphocytes was observed at 3 and 5 dpi (Figure 5A,B), but the profiles of these cells were different, mainly at 5 dpi (Figure 5C–F). Animals treated with Zi showed more Th17 cells than IL-10 producing CD4+ T cells at 3 dpi (Figure 5C). At 5 dpi, CD4+ T cells from untreated infected animals did not show differences among their populations (Figure 5E); however, CD4+ T cells from Zi-treated animals showed a higher number of Treg cells (FOXP3+ IL-10+) than their other cell subtypes (Figure 5E). Notably, the Zi treatment also resulted in significant increase in the CD8+ T-producing IL-10 and CD8+ Tregs (FOXP3+ IL-10+) cells, when compared with CD8+ T producers of IL-17 and IFN-γ (Figure 5F).

### 3.6. Zi Treatment Reduced the Number of Innate Immune Cells in the Spleen during MHV-3 Infection

**Figure 6:**
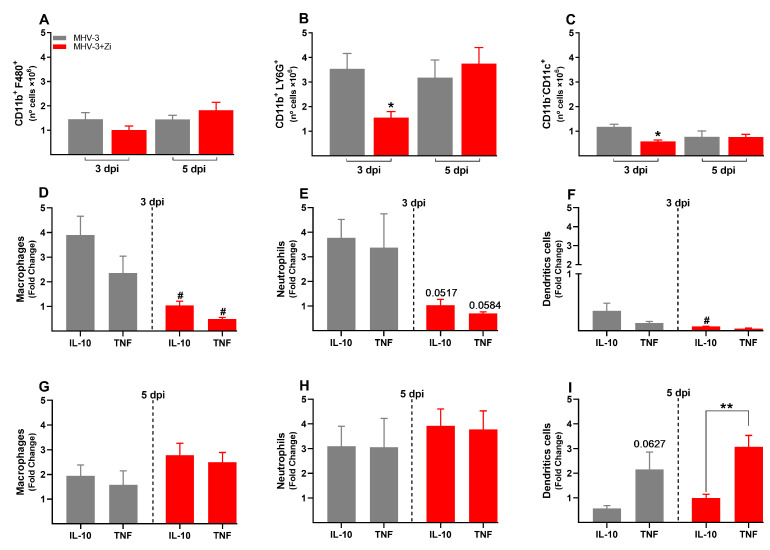
Flow cytometric analyses showing the total cell numbers in the spleen at 3 and 5 dpi among macrophages (CD11b+ F480+) (**A**), neutrophils (CD11b+ LY6G+) (**B**), and dendritic (CD11b-CD11c+) (**C**) groups. Macrophages (**D**,**G**), neutrophils (**E**,**H**), and dendritic cell-producing (**F**,**I**) IL-10 and TNF at 3 and 5 dpi. * *p* < 0.05; ** *p* < 0.01 (* representation among the same groups); (# representation between different groups). Abbreviations: IL, interleukin; TNF, tumor necrosis factor.

At 3 dpi, but not at 5 dpi, there was a reduced number of neutrophils and dendritic cells, but not macrophages, in the spleens of animals treated with Zi compared to that in the untreated infected group (Figure 6A–C). Notably, at 3 dpi, but not 5 dpi, treatment with Zi reduced the numbers of macrophages and neutrophils producing IL-10 and TNF, and dendritic cells producing IL-10 (Figure 6D–I).

### 3.7. Zi Treatment Modulates the Expansion/Generation of T Cells in the Spleen during MHV-3 Infection

**Figure 7:**
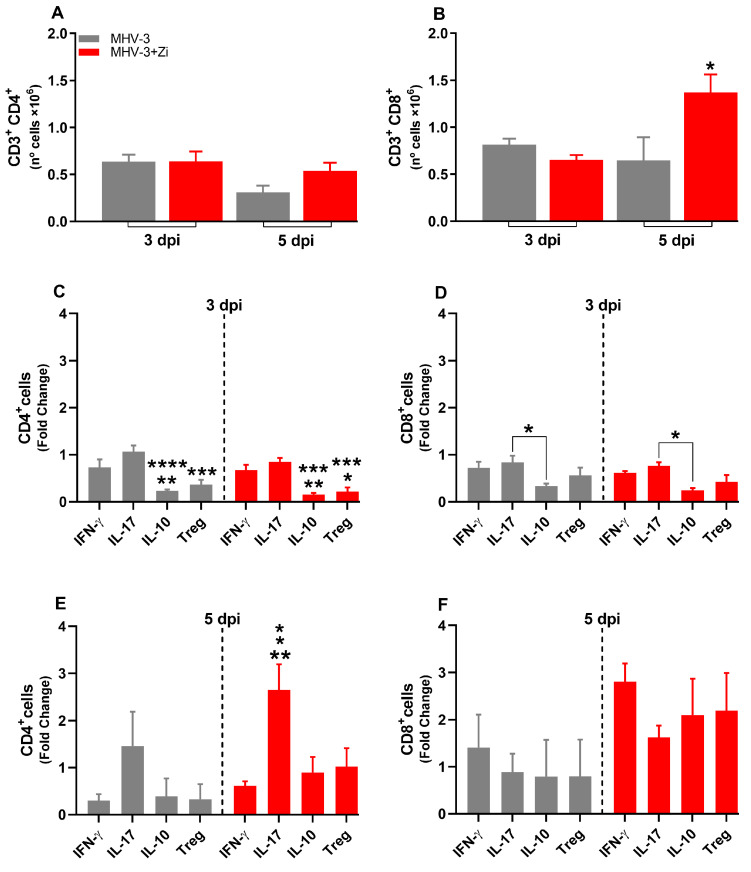
Flow cytometric analyses showing the total cell numbers in the spleen at 3 and 5 dpi between the CD3+CD4+ (**A**) and CD3+CD8+ (**B**) groups. CD4+ (**C**,**E**) and CD8+ (**D**,**F**) cells-producing IFN-γ, IL-17, IL-10, and Treg at 3 and 5 dpi. * *p* < 0.05; ** *p* < 0.01; *** *p* < 0.001; **** *p* < 0.0001. Abbreviations: IFN-γ, interferon-gamma; IL, interleukin.

In the spleen, at 3 dpi, there was no difference between the groups in the numbers of TCD4+ and TCD8+ lymphocytes (Figure 7A,B), whereas at 5 dpi, an increased number of CD8+ T cells was observed upon Zi treatment compared to that in the untreated infected group (Figure 7B). Checking the profile of these lymphocytes, we observed that at 3 dpi, the CD4+ T cells of both groups were more polarized to Th1 and Th17 profiles than to Th2 (IL-10) and Tregs (Figure 7C). Notably, among the CD8+ T cells in both groups, there were more IL-17 producers than IL-10 producers (Figure 7D). At 5 dpi, an increase in Th17 cells was observed in the treated group compared to the Th1, Th2, and Treg profiles (Figure 7E). However, no differences were observed in the CD8+ T cell profiles in either group (Figure 7F).

### 3.8. Zi Improves Clinical Parameters and Protects hK18ACE2 Mice from Lung Injury Induced by SARS-CoV-2

**Figure 8:**
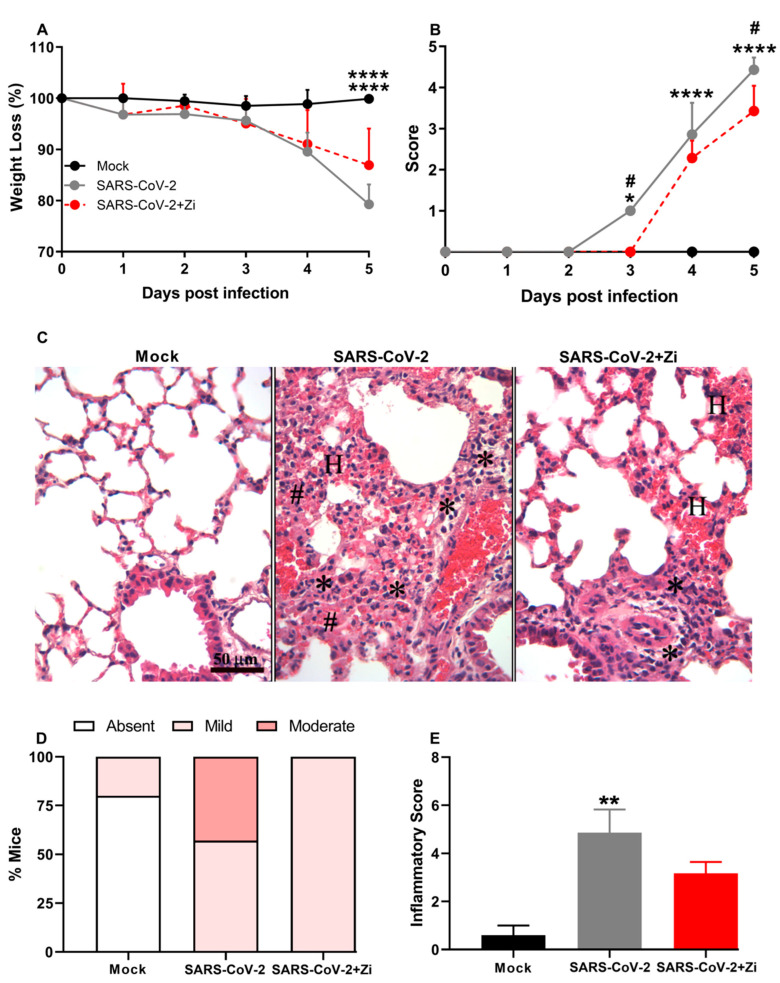
Body weight change after SARS-CoV-2 infection was evaluated using bidirectional repeated measures analysis of variance (ANOVA) and Sidak’s multiple comparison test. Data are presented as mean ± standard error of the mean (SEM) (**A**). Clinical scoring of control animals, mice infected with SARS-CoV-2, and infected and treated with zileuton (Zi), was performed daily until the end of the experiment (**B**). Hematoxylin and eosin (H&E) staining of lung sections showing signs of the inflammatory lesions in infected mice (**C**); * foci inflammatory, # hyperplasia and H hemorrhage. Percentages of mice according to the degree of inflammatory cell infiltration (**D**). Histopathological evaluation regarding the general inflammatory score. Comparisons between the sham and infection groups were performed using the Kruskal–Wallis and Dunn post hoc tests (**E**). * *p* < 0.05; ** *p* < 0.01; **** *p* < 0.0001. (* representation among the same groups); (# representation between different groups).

We validated the effects of Zi treatment in a translational model of SARS-CoV-2 infection. Therefore, we investigated the effects of Zi treatment on SARS-CoV-2 infection in transgenic animals expressing the human angiotensin I-converting enzyme 2 (ACE2) receptor. Treatment with Zi reduced weight loss and significantly improved the clinical score compared to untreated animals (Figure 8A,B). Notably, during coronavirus infection, treatment with Zi significantly reduced the inflammatory score in the lungs (Figure 8C,D), improving the histopathological pattern (Figure 8D), with a higher percentage of animals having mild lesions than the untreated group (Figure 8E).

## 4. Discussion

Ecosanoids or eicosanoids (from the Greek eikosi for “twenty”) are important lipid mediators for body homeostasis [27]. These mediators are oxygenated metabolites from the metabolism of Arachidonic Acid (AA), which acts at small concentrations on target cells, usually via G protein-coupled receptors (GPCRs) [28]. Eicosanoids were initially described for their ability to promote biological responses such as platelet aggregation, edema, and smooth muscle contraction; however, other functions, such as participation in inflammation, cancer, and immune responses, have been attributed to them [29]. Examples of eicosanoids include leukotrienes (LTs) and lipoxin (LXAs) [27]. LTs and LXAs have pro- and anti-inflammatory activities, respectively, and are produced via the lipoxygenase pathway, mainly involving the activity of 5-lipoxygenase enzyme (5-LO) [30].

Of great relevance, studies have shown that LTs play a key role in the development of respiratory distress syndrome (SDRA), which is knowing to be one of the most important clinical consequences of COVID-19 [8]. We and others have suggested that in SARS-CoV-2-mediated SDRA, agents such as 5-LO inhibitors could reduce the consequences of virus-induced cytopathic effects by immediate action on critical immune cells, which are associated with cytokine release syndrome in COVID-19 [14]. Thus, we sought to verify the effect of the selective 5-LO inhibitor, Zi, against infection in murine models of SDRA induction, which is extremely useful for studies of viral pathogenesis and antiviral therapy.

Initially, our results demonstrated that treatment with Zi improved clinical parameters and delayed the mortality of the animals, and a dose of 30 mg/kg promoted approximately 20% survival. The model used here was described by our group as a model of severe COVID-19, where 100% of the animals died, presenting with severe pneumonia and an increase in several cytokines in lung tissue [17]. Notably, the protection conferred by Zi in this model was of great relevance, mainly because we observed that this protection was not dependent on a reduction in viremia. Treatment with Zi did not protect the animals against leukopenia, lymphopenia, and thrombocytopenia caused by the virus; however, the treated animals did not present a reduction in granulocytes observed in the untreated group, suggesting that Zi is able to regulate the number of granulocytes during MHV-3 infection. Several studies have shown that uncontrolled inflammation is key to the severity of disease caused by SARS-CoV-2, this inflammation initially occurs in the lungs and can become systemic, causing shock, multiple organ failure, and death [1,2,3,4]. Here, we demonstrated that Zi significantly reduced the inflammatory process in the lungs caused by the murine coronavirus. In fact, Zi, being a selective 5-LO inhibitor, leads to the reduction in LTs, which are mediators with pro-inflammatory actions. LTs are divided into two classes: chemoattractant LTB4, which contains only hydroxyl fractions, and cysteinyl LTs (CysLTs: LTC4, LTD4, and LTE4), which contain amino acid fractions [31,32]. LTB4 is a potent monocyte-macrophage and neutrophil chemoattractant that is involved in T cell migration, increases the activity of dendritic cells, and promotes their migration by draining lymph nodes. It also increases TNF production and acts synergistically with IL-4 to activate B lymphocytes. LTC4, LTD4, and LTE4 induce tissue edema, mucus secretion and bronchoconstriction [14]. Blocking LTs caused a change in the cytokine expression profile in infiltrating cells of the lung tissue. At 3 dpi a greater proportion of IL-10, TNF, and IFN-γ was found; these cytokines play extremely important roles both in inflammatory processes and in the physiological processes of the body. TNF is an extremely important cytokine that has pleiotropic actions. In the inflammatory process, it regulates apoptosis, cell proliferation, and promotes the production of other chemokines and cytokines; in physiological processes, it becomes important for antitumor responses and homeostasis of the immune system; so, the presence of TNF, depending its levels, does not necessarily constitute a bad prognosis [33,34]. IL-10 is notably a cytokine known for its anti-inflammatory and Th1-type response inhibitory effects [35], while IFN-γ performs antiparasitic, antiviral, antitumor, and immunomodulatory functions, playing an important role in the immune response. In the innate immune system, IFN-γ-promotes the activation of macrophages (MOs), and in the adaptive immune response, it increases the proliferation of CD8+ T cells [36]. Notably, the presence of cytokines at the site of inflammation does not constitute a problem because they are necessary to contain the offending agent; however, their exacerbated production promotes deleterious effects, as observed in COVID-19 [3,37,38]. Some studies have shown that the balanced secretion of anti-inflammatory and pro-inflammatory cytokines, such as IL-10 and IL-6, is characteristic of asymptomatic infections, whereas in symptomatic infections, there is a predominance of pro-inflammatory cytokines [39,40,41]. As an exacerbated inflammatory process can cause damage to the host and loss of function in the affected tissue, we sought to observe whether the physiological functions of the infected animals would be affected. Remarkably, our results demonstrated that Zi treatment induces higher levels of IL-10 and IL-6 in the lungs.

As demonstrated by Andrade et al. in 2021 [17], infection with MHV-3 promotes an increase in the viral load in the cardiac tissue and damages the lung tissue, thereby reducing the compliance of the respiratory system. Compliance can be defined as the ability to expand, lower respiratory compliance, and become a more rigid system [42]. In this context, measuring the PV curve in mice offers a method for tracking the development of pathophysiological alterations over time and assessing the potential impact of therapeutic interventions [20,21,42]. Lung function can be altered by the fibrotic process that occurs after exaggerated inflammation, or by the extent of edema caused by acute inflammation [17,20]. In the present study, MHV-3 infection reduced respiratory compliance, as previously reported in an experimental model [17], and in human patients affected by COVID-19 [43]. Importantly, we observed maintenance of lung function in the Zi-treated group, in which compliance was not significantly decreased. The lower inflammatory score in the lung tissue of Zi-treated animals was likely responsible for the lack of impaired respiratory compliance.

Within the context of SARS-CoV-2 infection, various cardiac manifestations have been documented. These include atrial arrhythmias such as atrial fibrillation or atrial flutter, sinus node dysfunction, atrioventricular conduction irregularities, ventricular tachyarrhythmias, sudden cardiac arrest, and cardiovascular dysautonomia [44]. The etiology of these manifestations encompasses a spectrum of factors including direct viral intrusion, hypoxemia, localized and systemic inflammation, shifts in ion channel physiology, immune activation, and autonomic dysregulation [44].

Cardiac alterations significantly influence infection pathogenicity. Hospitalized COVID-19 patients with ventricular or atrial arrhythmias have an elevated risk of mortality, underscoring the clinical importance of these findings [45]. Consequently, the milder cardiac alterations observed in the treated animals serve as an additional indication of the efficacy of Zi in protecting against infection.

For the course of infection to occur in such a way that the virus is eliminated without extreme damage to the host, it is necessary that the innate immune response is robust enough to eliminate the pathogen, but balanced to the point of not being deleterious to the tissue [4]. The innate immune response is known to provide the first line of defense against parasitic, viral, and bacterial infections. Among the cells of the innate immune response, neutrophils are among the first to migrate to the site of infection [46]. Studies have shown that neutrophils are increased in the nasopharyngeal epithelium and bronchoalveolar fluid of patients with severe COVID-19, and robust evidence suggests that these patients have elevated levels of extracellular neutrophil traps (NETs) in the plasma, serum aspirates from the trachea and lungs, and lung biosia [47,48,49], suggesting that neutrophils can play a critical role in this infection [3]. Here, treatment with Zi promoted an increase in IL-10-producing neutrophils, suggesting that the drug promotes the polarization of NEs towards a more anti-inflammatory profile, which may be one of the mechanisms by which it protects the lung tissue. Along with the increase in IL-10-producing NEs, was observed a reduction in TNF-producing DCs, a fact that could influence the presentation and activation of T lymphocytes, since DCs play a fundamental role in the connection between innate and adaptive immune responses. No major changes were observed in the innate immune response at 5 dpi, probably because this part of the immune system is more prominent at the beginning of the infection (3 dpi). In contrast, significant changes in adaptive immune responses were observed at 5 dpi. Adaptive immune response cells, CD4+ and CD8+ T cells, play crucial roles in the antiviral response. CD4+ T cells can differentiate into several subsets (Th1, Th2, Th17, and Treg are examples) and help CD8+ T cells release cytokines that activate and recruit more cells of the innate immune response, improve antiviral activities, and facilitate tissue repair, thus presenting functionality in broad aspects of the immune response [50]. We observed that the treatment did not cause differences in the number of CD4+ T cells in the lung at 3 and 5 dpi; however, at 3 dpi, there was an increase in IL-17 producing CD4 T cells (Th17) compared to IL-10 producing cells. Th17 cells play a variety of roles, including antimicrobial protective responses; maintenance of barriers in the skin, intestine, and lungs; and recruitment of NEs by stimulating the secretion of cytokines such as CXCL1 [51]. Interestingly, IL-17 has been associated with the presence of several pulmonary pathogens, which led us to believe that its presence in this infection is a precise way to delay viral dissemination. However, it is important to note that excess IL-17 could cause damage to lung tissue, as this cytokine promotes cell migration; thus, a fine adjustment to avoid exacerbating the proinflammatory response would be beneficial. Our data showed that Zi promotes an increase in IL-10, a cytokine with great anti-inflammatory potential, at 3 dpi; therefore, it is likely that Zi promotes this balance between cytokines to improve lung tissue and prevent viral perpetuation, which seems to be reflected in the reduction in the inflammatory score, maintenance of the pulmonary tissue, and clinical improvement observed here. Treatment with Zi promoted a change in the profile of CD4 + and CD8 + lymphocytes at 5 dpi, increasing the number of IL-10-and FOXP3+IL-10+ (Treg)-producing cells, which are thought to promote the regulation of the immune response [52], and is probably one reason for the increased survival observed in the treated group.

Seeking to observe organs involved in the generation/expansion of immune response, was found that the profile of the immune response in the spleen differs between infected and treated infected animals. At 3 dpi, the innate immune cells, mainly NE cells, were reduced in the treated animals, suggesting that these cells migrated to the target organ of the infection. Notably, increased levels of chemokines and cytokines were observed, which are important for the recruitment of cells to infected tissues. However, at 5 dpi, it was observed that treatment increased the number of CD8+ T-cells in the spleen. Our collaborators [17] demonstrated that at 5 dpi, the virus was already present in the bloodstream, and several studies have demonstrated that CD8+ T cells are important for viral containment [53]. In COVID-19, virus-specific CD8+ T lymphocytes are associated with better prognoses; therefore, this increase evidenced by us could be beneficial for viral containment [54].

Finally, it was observed that Zi also influences lung inflammation caused by SARS-CoV-2, showing that treatment with this drug improves the adjustment of the inflammatory response during infection with different strain of SARS-CoV-2 virus.

## 5. Conclusions

Our findings showed that the 5-LO inhibitor, Zi, modulates local and systemic immune responses, restrains pulmonary inflammation, ameliorates clinical disease, and enhances survival rates without interfering with the host’s ability to deal with MHV-3 and betacoronavirus-induced SARS-CoV-2 infections. Finally, evaluating whether the combination of Zi with an antiviral drug is an ideal treatment for coronavirus infection is important.

## Data Availability

Data are available upon request.
